# SHEL5K: An Extended Dataset and Benchmarking for Safety Helmet Detection

**DOI:** 10.3390/s22062315

**Published:** 2022-03-17

**Authors:** Munkh-Erdene Otgonbold, Munkhjargal Gochoo, Fady Alnajjar, Luqman Ali, Tan-Hsu Tan, Jun-Wei Hsieh, Ping-Yang Chen

**Affiliations:** 1 Department of Computer Science and Software Engineering, College of Information Technology, United Arab Emirates University, Al Ain 15551, United Arab Emirates; omunkuush@uaeu.ac.ae (M.-E.O.); fady.alnajjar@uaeu.ac.ae (F.A.); 201990024@uaeu.ac.ae (L.A.); 2 RIKEN Center for Brain Science (CBS), Wako 463-0003, Japan; 3 Department of Electrical Engineering, National Taipei University of Technology, Taipei 10608, Taiwan; thtan@ntut.edu.tw; 4 College of Artificial Intelligence and Green Energy, National Yang Ming Chiao Tung University, Hsinchu 30010, Taiwan; jwhsieh@nctu.edu.tw; 5 Department of Computer Science, National Yang Ming Chiao Tung University, Hsinchu 30010, Taiwan; pingyang.cs08g@nctu.edu.tw

**Keywords:** YOLOv3, YOLOv4 YOLOv5, YOLOR, safety helmet, SHEL5K, object detection, benchmark dataset

## Abstract

Wearing a safety helmet is important in construction and manufacturing industrial activities to avoid unpleasant situations. This safety compliance can be ensured by developing an automatic helmet detection system using various computer vision and deep learning approaches. Developing a deep-learning-based helmet detection model usually requires an enormous amount of training data. However, there are very few public safety helmet datasets available in the literature, in which most of them are not entirely labeled, and the labeled one contains fewer classes. This paper presents the Safety HELmet dataset with 5K images (SHEL5K) dataset, an enhanced version of the SHD dataset. The proposed dataset consists of six completely labeled classes (*helmet*, *head*, *head with helmet*, *person with helmet*, *person without helmet*, and *face*). The proposed dataset was tested on multiple state-of-the-art object detection models, i.e., YOLOv3 (YOLOv3, YOLOv3-tiny, and YOLOv3-SPP), YOLOv4 (YOLOv4 and YOLOv4_pacsp-x-mish_), YOLOv5-P5 (YOLOv5s, YOLOv5m, and YOLOv5x), the Faster Region-based Convolutional Neural Network (Faster-RCNN) with the Inception V2 architecture, and YOLOR. The experimental results from the various models on the proposed dataset were compared and showed improvement in the mean Average Precision (mAP). The SHEL5K dataset had an advantage over other safety helmet datasets as it contains fewer images with better labels and more classes, making helmet detection more accurate.

## 1. Introduction

Workplace safety has become a focus for many production and work sites due to the consequences of the unsafe environment on the health and productivity of the workforce. According to statistics [1,2,3,4], the construction industry is at high risk for the injuries and deaths of workers. In 2005, the National Institute for Occupational Safety and Health (NIOSH) reported 1224 deaths of construction workers in 1 y, making it the most dangerous industry in the United States (U.S.) [1]. Moreover, the U.S. Bureau of Labor Statistics (BLS) estimated injuries for 150,000 workers every year at construction sites [1]. The Bureau also reported the death of one in five workers in 2014 and a total of 1061 construction workers’ deaths in 2019 [2,3]. As per the report of the Ministry of Employment and Labor (MEOL) in Korea, 964 and 971 workers died in workplace accidents in 2016 and 2017, respectively [4]. Among these fatalities, 485 fatalities occurred at construction sites, followed by 217 and 154 in the manufacturing and service industry, respectively. Workers at most of the worksites and manual working environments are at high risk of injuries because of not following the safety measures and using Personal Protective Equipment (PPE). The carelessness of the workers and not following PPE compliance will have adverse effects and pose more threats of minor or major injuries. In 2012, the National Safety Council (NSC) reported more than 65,000 cases of head injuries and 1020 deaths at construction sites [5]. According to the American Journal of Industrial Medicine, a total number of 2210 construction workers died because of a Traumatic Brain Injury (TBI) from 2003 to 2010 [6]. Released by Headway, the brain injury association, 3% of PPE purchased was for head protection as head injuries account for more than 20% of total injuries [7].

These statistics delineate the prevalence of fatal and non-fatal injuries in the construction industry, and there is a dire need to reduce the rate. Creating a safe environment for workers brings an arduous challenge for this sector globally. Adopting safety measures and providing construction workers with PPE can result in decreasing accident rates. Despite the effectiveness of these strategies, it is not guaranteed that the workers would be cautious and use the PPE. To avert all these troubles, there is a need to discover automated ways of detection and monitoring safety helmets. A deep-learning-based safety helmet detection system can be developed by using a large amount of labeled data. However, there is a lack of datasets to build highly accurate deep learning models for workers’ helmet detection. There are few publicly available datasets for safety helmet detection, which are not entirely labeled, and the labeled ones contain fewer classes and incomplete labels. Therefore, the proposed work presents the Safety HELmet dataset with 5K images (SHEL5K) dataset, an enhanced version of the SHD dataset [8]. In the SHD dataset [8], many objects are not labeled, which is not sufficient to train an efficient helmet recognition model. The SHD dataset [8] was improved in the proposed work by labeling all three originally proposed classes and adding three more classes for training an efficient helmet detection model. The main aims of the proposed study were to: (1) complete the missing labels and (2) increase the number of classes from three to six (*helmet*, *head with helmet*, *person with helmet*, *head*, *person without helmet*, and *face*). The proposed dataset was tested on various object detection models, i.e., YOLOv3 [9], YOLOv3-tiny [10], YOLOv3-SPP [11], YOLOv4 [12], YOLOv5-P5 [13], the Faster Region-based Convolutional Neural Network (Faster-RCNN) [14] with the Inception V2 architecture [15], and YOLOR [16] models. The experimental results showed significant improvements in the mAP as compared to the publicly available datasets. A comparative analysis was performed, and discussions are provided based on results from the various models. The proposed system was also used to successfully perform real-time safety helmet detection in YouTube videos.

## 2. Related Work

In the literature, various efforts have been made by researchers to develop a vision-based system for the helmet detection task. Li et al. [17] proposed a Convolutional-Neural-Network (CNN)-based safety helmet detection method using a dataset of 3500 images collected by the web crawling method. The precision and recall of the system were recorded as 95% and 77%, respectively. Wang et al. [18] proposed a safety helmet detection model trained on a total of 10,000 images captured by 10 different surveillance cameras at construction sites. In the experiment’s first phase, the authors employed the YOLOv3 architecture [9] and achieved an *mAP*0.5 of 42.5%. In the second phase, the authors improved the architecture of YOLOv3 [18] and achieved an *mAP*0.5 of 67.05%. Wang et al. [19] suggested a hardhat detection system based on a lightweight CNN using the Harvard database hardhat dataset [20]. The dataset contains 7064 annotated images, which consist of three classes (*helmet*, *head*, and *person*). In the three classes, the *person* class is not appropriately labeled. The network was trained considering two classes (*helmet* and *head*) and achieved an average accuracy of 87.4% and 89.4% for *head* and *helmet*, respectively. Li et al. [21] trained an automatic safety helmet-wearing detection system using the INRIA person dataset [22] and collected pedestrian data from a power substation. The authors in [21] showed that the accuracy of the proposed method (Color Feature Discrimination (CFD) and the ViBE algorithm in combination with the c4 classifier) yielded better results than HOG features and the SVM classifier method. The accuracy of the HOG feature with the SVM classifier achieved 89.2%, while the proposed method achieved an accuracy of 94.13%. Rubaiyat et al. [23] proposed an automated system for detecting helmets in construction safety. The authors collected 1000 images from the Internet using a web crawler, which consisted of 354 human images and 600 non-human images. The helmet class achieved an accuracy of 79.10%, while the without helmet class achieved an accuracy of 84.34%. Similarly, Kamboj and Powar [24] proposed an efficient deep-learning-based safety helmet detection system for the industrial environment by acquiring data from various videos of an industrial facility. The videos were captured by using cameras having a resolution of 1920 × 1080 px and a frame rate of 25 frames per second. The dataset consisted of 5773 images having two classes (*helmet* and *without helmet*). An improved helmet detection was proposed by Geng et al. [25] using an imbalanced dataset of 7581 images, mostly with a person in a helmet and a complex background. The label confidence of 0.982 was achieved by testing it on 689 images. Moreover, Long et al. [26] proposed a deep-learning-based detection of safety helmet wearing using 5229 images, acquired from the Internet and various power plants (including power plants under construction). The proposed system was based on SSD, and an *mAP*0.5 of 78.3% was achieved on the test images and compared with SSD, which was 70.8% using an IoU of 0.5. In the above studies [17,18,23,24,26], they used custom data to test their method; therefore, it is not fair to make a comparison of the proposed work in this paper with these methods.

### 2.1. Datasets for Safety Helmet Detection

In general, researchers develop helmet detection systems using custom data or publicly available datasets. Some of the publicly available datasets, i.e., [8,20,27,28], for safety helmet detection are summarized in Table 1. Table 1 shows a brief comparison of the proposed dataset in the current study with various publicly available datasets. Each dataset shown in Table 1 is explained in detail below.

#### 2.1.1. Safety Helmet Detection Dataset

**Figure 1:**
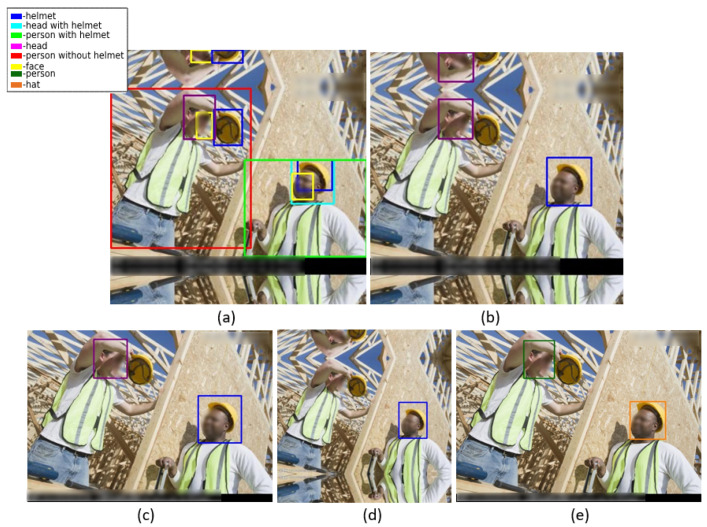
Comparison of public safety helmet datasets’ labels and SHEL5K dataset’s labels: (**a**) SHEL5K dataset, (**b**) SHD dataset [8], (**c**) hardhat dataset [20], (**d**) HHW dataset [27], and (**e**) SHW dataset [28].

The Safety Helmet Detection (SHD) dataset [8] is a publicly available dataset on Kaggle containing 5000 labeled images and three classes (*helmet—18,966*, *head—5785*, and *person—751*). However, the dataset has many incompletely labeled objects. Figure 1b shows the dataset labels, which shows that the *person* class is not labeled.

#### 2.1.2. Hardhat Dataset

The hardhat dataset [20] is a safety helmet dataset shared by Northeastern University consisting of 7063 labeled images. The dataset is divided into training and testing sets, which contain 5297 and 1766 images, respectively. The images are from three distinct classes having 27,249 labeled objects (*helmet—19,852*, *head—6781*, and *person—616*). In the given dataset, the *person* class is not labeled properly, as shown in Figure 1c, and the number of images in each class is not distributed equally.

#### 2.1.3. Hard Hat Workers Object Detection Dataset

The Hard Hat Workers (HHW) dataset [27] is an improved version of the hardhat dataset [20] and is publicly available on the Roboflow website. In the HHW dataset [27], the number of labels in each class is increased (*helmet—26,506*, *head—8263*, and *person—998*). Figure 1d shows a sample image of the HHW dataset [27] labels in which it can be seen that the *person* class is not labeled.

#### 2.1.4. Safety Helmet Wearing Dataset

The Safety Helmet Wearing (SHW) dataset [28] consists of 7581 images. The images have 111,514 safety helmet-wearing or positive class objects and 9044 not-wearing or negative class objects. Some of the negative class objects were obtained from the SCUT-HEAD dataset [29]. Several bugs of the original SCUT-HEAD dataset [29] were fixed to directly load the data into a normal PASCAL VOC format. Most images in the dataset are helmet images, and there are a very small number of head images. Figure 1e shows a labeled sample image from the SHW dataset. Figure 1a shows a comparison between the public datasets’ labels and the SHEL5K dataset’s labels.

## 3. SHEL5K Dataset

**Figure 2:**
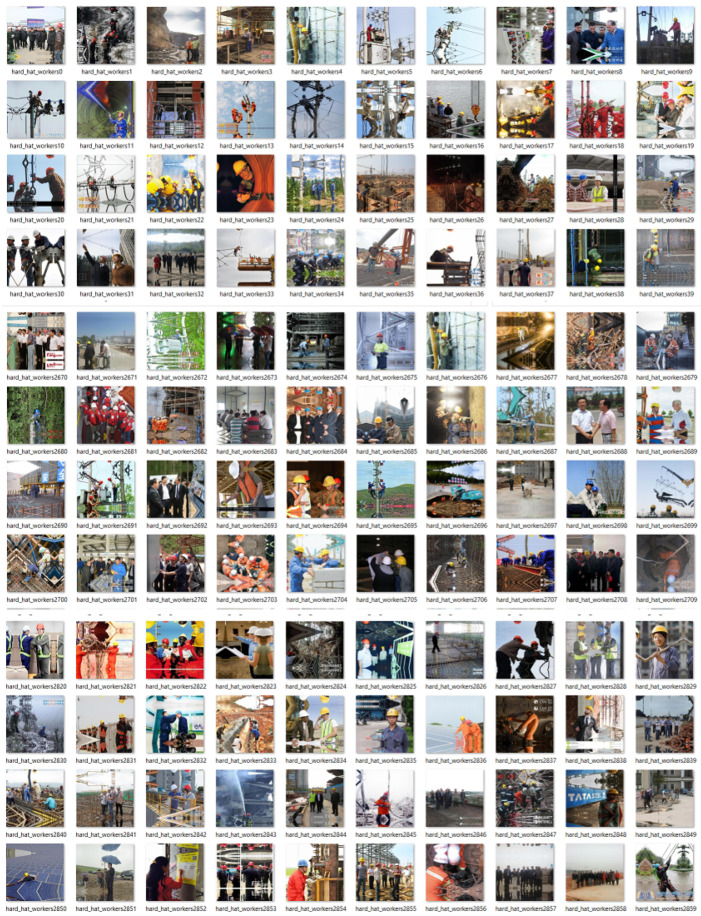
Sample images of the SHEL5K dataset.

**Figure 3:**
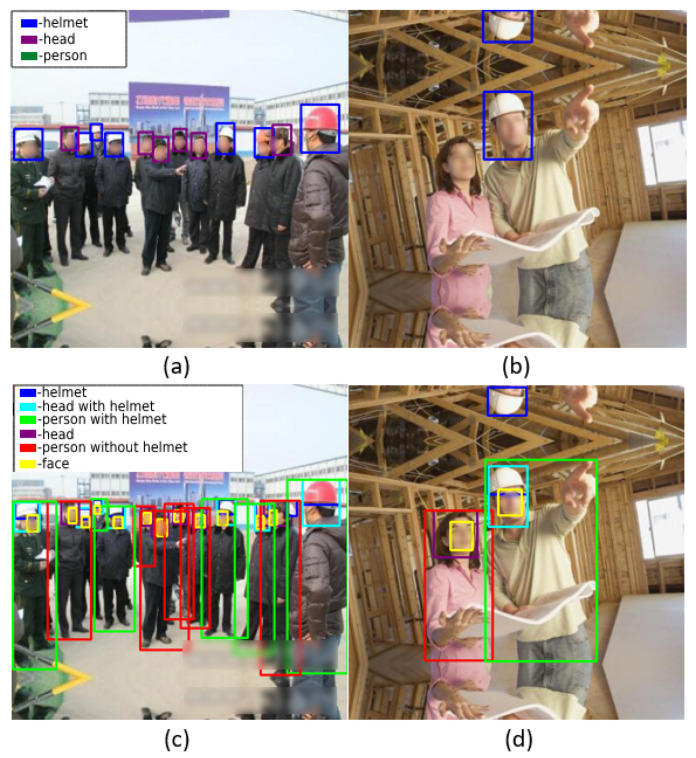
(**a**,**b**) SHD dataset [8] labels; (**c**,**d**) SHEL5K dataset labels.

In the proposed work, the number of labels and classes in the SHD dataset [8] were extended and completed. Figure 2 shows sample images of the SHD dataset [8]. The SHD dataset [8] contains 5000 images having a resolution of 416 × 416 and 25,501 labels with complicated backgrounds and bounding box annotations in PASCAL VOC format for the three classes namely *helmet*, *head*, and *person*. The limitation of the SHD dataset [8] is that numerous objects are incompletely labeled. Figure 3a,b shows image samples with *person* and *head* not properly labeled. The main aims of the proposed study were to: (1) completed the missing labels and (2) increase the number of classes from three to six (*helmet*, *head with helmet*, *person with helmet*, *head*, *person without helmet*, and *face*).

**Figure 4:**
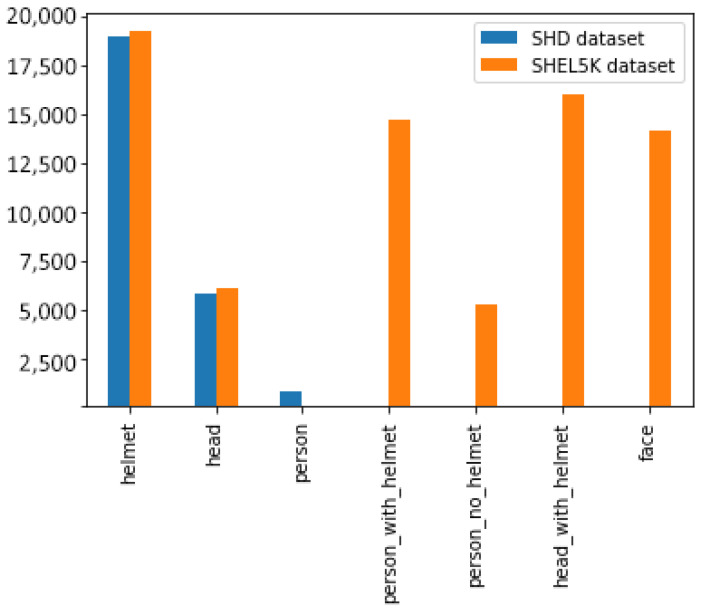
Bar graph comparison between the SHD dataset [8] and SHEL5K dataset in terms of the number of labels for each class.

To address the limitations associated with the SHD dataset, SHEL5K is proposed, which consists of 75,570 labels. The number of labels in the SHEL5K dataset was increased for each class, i.e., (*helmet—19,252*, *head—6120*, *head with helmet—16,048*, *person without helmet—5248*, *person with helmet—14,767*, and *face—14,135*). Figure 3 shows the comparison of the labels of the SHD dataset [8] (a and b) and SHEL5K datasets (c and d), with the helmet in blue, the head in purple, the head with helmet in navy blue, the person with helmet in green, the person without a helmet in red, and the face in the yellow bounding boxes. Moreover, the graph in Figure 4 shows the comparison of the SHD dataset [8] and SHEL5K dataset in terms of the number of labels of each class. The SHD dataset [8] and SHEL5K labels are represented by blue and orange bars, respectively. From the graph, it can be seen that the class *person* is too poorly labeled. In the proposed work, the labeling of the image was performed by using the LabelImg [30] tool with the following steps: (1) the default number of classes in the tool was changed to six for our dataset; (2) images opening and label saving paths were specified; (3) objects corresponding to the classes were labeled, and an XML file was created.

The file contains the name of the image, the path to the image, the image size and depth, and the coordinates of the producer image.

## 4. Results and Discussion

The proposed dataset SHEL5K was benchmarked by using state-of-the-art one-stage object detection models such as YOLOv3 [9], YOLOv4 [12] YOLOv5-P5 [13], the Faster-RCNN [14] with Inception v2 [15], and YOLOR [16]. In particular, we employed different pretrained variations of the models, i.e., YOLOv3-tiny [10], YOLOv3 [9], YOLOv3-SPP [11], YOLOv3-SPP pretrained on the MS COCO dataset [31], YOLOv3-SPP pretrained on the ImageNet dataset [32], and YOLOv5-P5 models (YOLOv5s, YOLOv5m, YOLOv5x) [13]. These models were prepared using the COCO 128 dataset, which contains the first 128 images of COCO train 2017 [31].

### 4.1. Evaluation Metrics

In the proposed work, the precision, recall, F1 score, and *mAP* were used as the evaluation metrics to perform a fair comparison between the experimental results of the models. The precision represents the object detection model’s probability of the predicted bounding boxes being identical to the actual ground truth boxes and is described in Equation (1) below.

(1) $$\mathrm{Precision}=\frac{TP}{\left(TP+FP\right)}$$

where *TP*, *TN*, *FP*, and *FN* refer to True Positive, True Negative, False Positive, and False Negative, respectively. The recall represents the probability of ground truth objects being correctly detected as depicted in (2).

(2) $$\mathrm{Recall}=\frac{TP}{\left(TP+FN\right)}$$

Moreover, the F1 score is the harmonic mean of the model’s precision and recall, and the mathematical representation is shown in Equation (3).

(3) $$F1\;\mathrm{score}=2*\frac{\mathrm{Precision}*\mathrm{Recall}}{\mathrm{Precision}+\mathrm{Recall}}$$

Additionally, the mean Average Precision (*mAP*) is the score achieved by comparing the detected bounding box to the ground truth bounding box. If the intersection over union score of both the boxes is 50% or larger, the detection is considered as *TP*. The mathematical formula of the *mAP* is given in Equation (4) below.

(4) $$mAP=\frac{1}{n}\sum_{k=1}^{k=n}AP_{k}$$

where $AP_{k}$ is the average precision of class *k* and *n* represents the number of classes.

### 4.2. Experimental Setup

Data preparation started with the conversion of annotated files from the PASCAL VOC format to the YOLO format to be given as the input to object detection models. The proposed dataset was randomly divided into training and testing sets. The training set contained a total of 4000 (80%) images, while the testing set contained 1000 (20%) images. The criterion for evaluating the performance of the various models were the *mAP*0.5 (the and F1 score. During the experiments, the Intersection over Union (IoU) threshold value was kept at 0.5. YOLOv3-SPP [11], YOLOv4 [12], YOLOv5-P5 [13], and YOLOR [16] were considered trained on the proposed dataset as these models have the fastest inference time for real-time object detection as compared to the majority of object detection models. The reason is that these models perform classification and bounding box regression in a single step. Empirically, it was found that the suitable number of epochs for training the YOLOv3 models (YOLOv3-tiny [10], YOLOv3 [9], and YOLOv3-SPP [11]), and the Faster-RCNN [14] with Inception v2 [15] was 1000, while for the other models, namely YOLOv4 [12], YOLOv5-P5 [13], and YOLOR [16], the value was 500. The performance of these models was also compared with the Faster-RCNN [14] with Inception v2 [15] model, which is better at detecting small objects. The Faster-RCNN [14] with Inception V2 [15] model was trained with 250,000 steps, and the learning rate was set to 0.0002 while keeping the value of the batch size equal to 16. The results of the Faster-RCNN [14] with Inception v2 [15] were measured using the comparative analysis of object detection metrics with a companion open-source toolkit [33], which is similar to the YOLO models.

### 4.3. Three-Class Results

**Figure 5:**
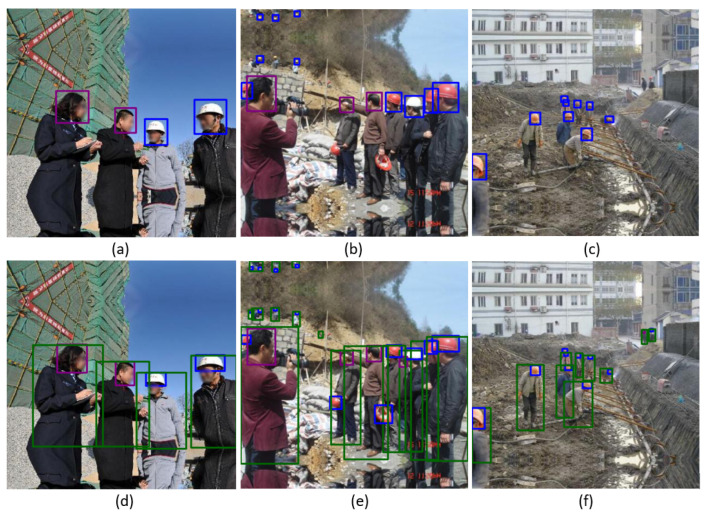
Comparison of the SHD and SHEL5K dataset results on the same images. (**a**–**c**) The results of the best SHD dataset [8] model. (**d**–**f**) The results of the best SHEL5K dataset model.

The SHD dataset [8] has three classes (*helmet*, *head*, and *person*) and the SHEL5K dataset has six classes (*helmet*, *head with a helmet*, *a person with a helmet*, *head*, *person without a helmet*, and *face*). In the current study, two classes *person with helmet* and *person without helmet* were combined to perform a fair comparison between the two datasets. Both classes were merged, as they correspond to the class *person* in the SHD dataset [8]. Figure 5 shows the comparison of the SHD and SHEL5K dataset results on the same images. For the SHD dataset [8] results, the person class was not detected. Table 2 shows the comparison results of the YOLOv3-SPP [11] and YOLOv5x [13] models on the SHD dataset [8] and SHEL5K dataset. For the sake of simplicity, the results of two models, YOLOv3-SPP [11] and YOLOv5x [13], are presented as they outperformed the remaining models. For the YOLOv5x [13] model, an *mAP*0.5 of 0.8528 was achieved for three classes, where the best and worst *mAP*0.5s were 0.8774 and 0.8311 for the *helmet* and *person* classes, respectively. The trained model showed low performance in the case of the *person* class in comparison with the *helmet* class. The YOLOv5x [13] model achieved better performance than YOLOv3-SPP [11] as shown in Table 2. The head class in the SHD dataset [8] achieved high a precision, recall and F1 score as it was properly labeled in comparison with the other classes. The helmet class results did not perform well as head with helmet and helmet were given a single label helmet in the dataset. Moreover, the results of the person class were low as the labeling of the person class was incomplete in the SHD dataset [8].

**Figure 6:**
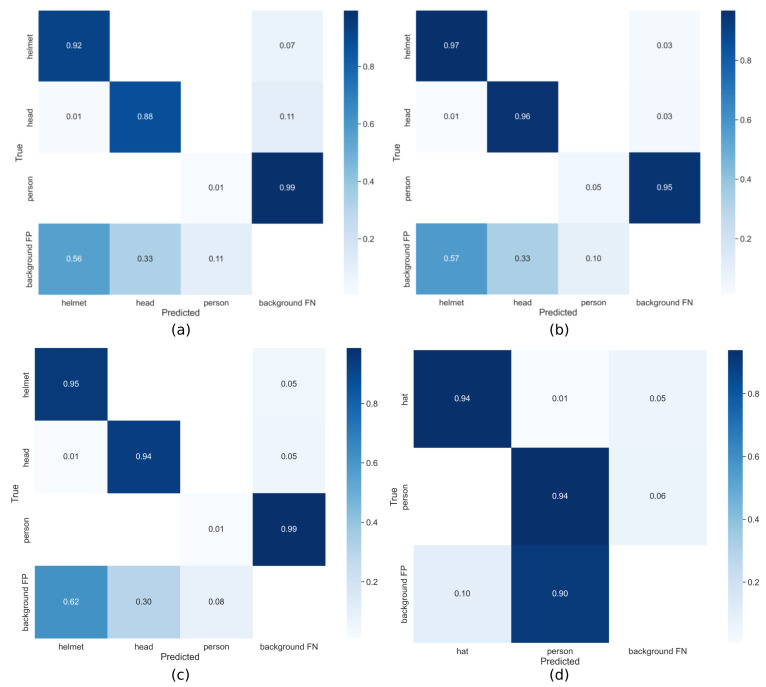
Confusion matrices of the YOLOv5x model on (**a**) the SHD dataset [8], (**b**) hardhat dataset [20], (**c**) HHW dataset [27], and (**d**) SHW dataset [28].

Figure 6 shows the confusion matrices for the YOLOv5x [13] model based on various publicly available datasets. The confusion matrices computed for the HHW dataset [27], the hardhat dataset [20], and the SHD dataset [8] for three classes (*helmet*, *head*, and *person*) are plotted in Figure 6a–c, respectively. The confusion matrices showed very poor results for the class *person*, and the background FNs were also high. The background FNs are the unrecognized percentage of the labeled object, and its results were for all three datasets. The *helmet* and *head* classes performed very well, and the background FPs were recorded as high. Figure 6d shows the confusion matrices constructed by the object detection models on the SHW dataset [28] test dataset for two classes (*hat* and *person*). The confusion matrix shows that the YOLOv5x [13] model showed good performance on the SHW dataset [28] as compared to the other datasets. Overall, the performance of the other datasets was also promising, except for the person class, which was not detected by the model. Therefore, in the current study, the dataset was extended and labeled properly, and additional classes were added to have a more accurate detection of the *person* class in the SHEL5K dataset.

### 4.4. Six-Class Results

**Figure 7:**
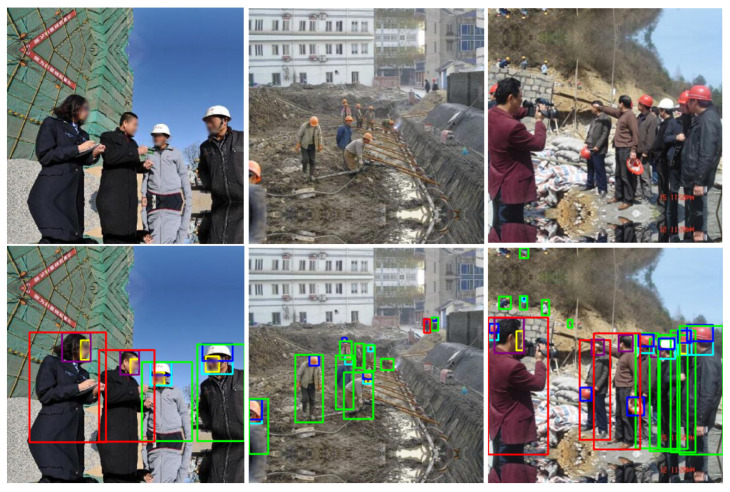
The YOLOv5x [13] detected outputs are plotted with the original images.

Table 3 and Table 4 show the comparison results of different variations of the YOLOv3-SPP [11] and YOLOv5-P5 [13] models trained on the SHEL5K dataset. For YOLOv3-SPP [11], three different models were evaluated using the SHEL5K dataset, in which one was trained from scratch (not pretrained) and the other two models were pretrained on the ImageNet dataset [32] and MS COCO dataset [31]. The highest *mAP*0.5 of 0.5572 was achieved by the YOLOv3-SPP [11] model pretrained on the ImageNet dataset [32]. For YOLOv3-SPP [11], the highest *mAP*0.5 of 0.6459 was achieved for the head with *helmet* class, while the two worst *mAP*0.5s of 0.007 and 0.0295 were reported for the *face* class when the model was trained from scratch and YOLOv3-SPP [11] was pretrained on the MS COCO dataset [31]. These models achieved an *mAP*0.5 value of nearly zero, which may be because the human faces were far away in most images and there was no face class included in the COCO dataset [31]. For the YOLOv5-P5 [13] model, Table 4 shows a comparison of the results of the three models of YOLOv5-P5 [13] on the SHEL5K dataset. The YOLOv5-P5 [13] model is available in the YOLOv5s, YOLOv5m, YOLOv5l, and YOLOv5x models. However, in the current study, the YOLOv5s, YOLOv5m, and YOLOv5x models on the pretrained COCO128 dataset [31] were selected. The YOLOv5x [13] achieved an *mAP*0.5 of 0.8033, and the class with the highest *mAP*0.5 of 0.8565 was the class *person with helmet*. The results of the *face* class were relatively poor, and the *mAP*0.5 was 0.7196. The *mAP*0.5 of YOLOv5-P5 [13] was better than YOLOv3-SPP [11]. The results of the YOLOv5x [13] model on three different types of images captured at various distances (far, near, and medium) are shown in Figure 7.

**Figure 8:**
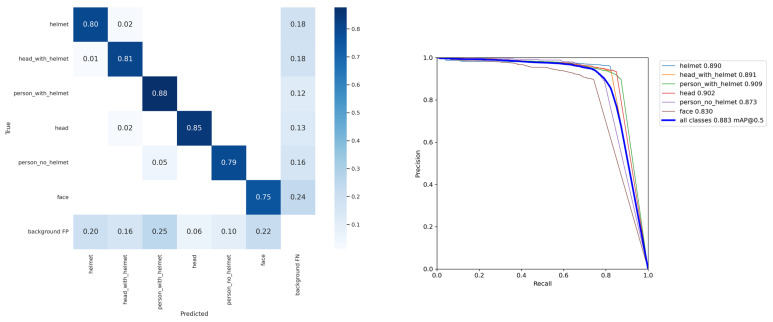
Confusion matrix and PR curve of the object detection model calculated on the SHEL5K dataset and the YOLOv5x [13] model.

Figure 8 shows the confusion matrix of the SHEL5K dataset. The results were relatively low compared to the other public datasets. This is also evident from Table 5, which shows the comparison results of the YOLOv5x [13] model on various datasets including the SHEL5K dataset. The model trained on the SHEL5K dataset showed better results compared to the other datasets except for the SHW dataset [28]. The precision, recall, and F1 score achieved by the model on the proposed dataset were slightly lower than the SHW dataset [28]. The precision, recall, and F1 score of the model on the SHEL5K were recorded as 0.9188, 0.817, and 0.8644, respectively. This is because the SHW dataset [28] contains only two classes, while the proposed dataset contains six classes. Moreover, during the labeling of the proposed dataset, an image containing some part of the helmet and face was labeled as the helmet or face class, respectively. The Precision–Recall (PR) curve is also shown in Figure 8, which also depicts that the lowest *mAP*0.5 (0.72) was achieved by the face class, which was less than the *mAP* (0.80) of all the other classes.

**Figure 9:**
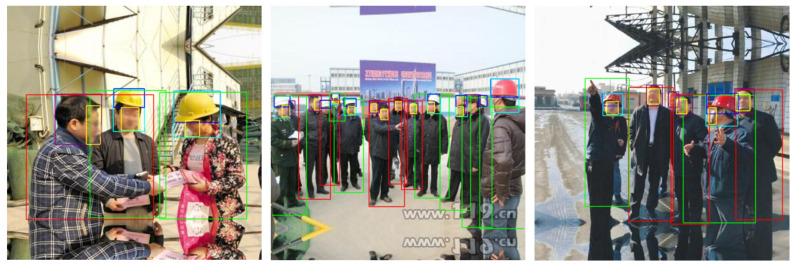
Result of the YOLOR [16] model experiments on the sample images.

The results of the YOLOR model on the proposed dataset (SHEL5K) are summarized in Table 6. The YOLOR [16] model used in the proposed work was pretrained on the COCO dataset [31]. The model achieved an *mAP*0.5 of 0.8828, and the highest *mAP*0.5 of 0.911 was recorded for the class *head with helmet*. The result of the class *person without helmet* was relatively poor with an *mAP*0.5 of 0.8498. The results of the model on the sample images are depicted in Figure 9.

**Figure 10:**
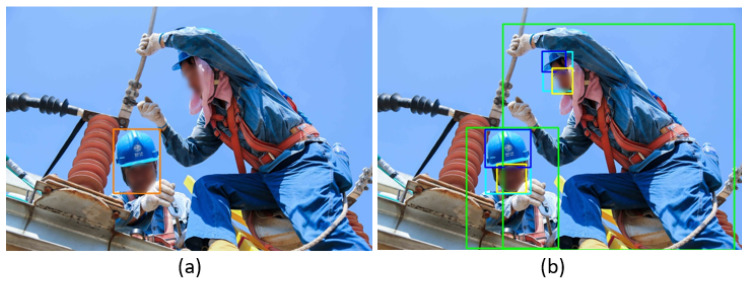
Comparison of the best-trained model results on the (**a**) SHW dataset [28] and the (**b**) SHEL5K dataset.

Figure 10 compares the visualization results of the best model trained on the SHW dataset [28] and the SHEL5K dataset on a test image. It can be seen from the result of the model trained on the SHW dataset [28] in Figure 10a that the model was not able to detect the *helmet* class if the helmet in the image was half visible and the head of the worker was hidden, as shown in Figure 10a. The results of the model trained on the SHEL5K dataset are shown in Figure 10b, which shows that the model can detect the *helmet* class correctly, which shows that the labeling in the proposed dataset was performed efficiently. The state-of-the-art model trained on the SHEK5K dataset in the current study did not perform well. However, in the future, the proposed dataset will be given to new object detection models to achieve high performance.

The K-fold cross-validation method was used to check whether the models were subjected to overfitting on the proposed data or not. The proposed dataset was divided into training 80% (4000 images) and testing 20% (1000 images). The value of K was considered five where the data were split into five folds, i.e., K1, K2, K3, K4 and K5. Table 7 shows the results of K-fold cross-validation on the SHEL5K dataset using the YOLOR [16] model. The results of all the folds were comparable, which shows that the model was not subjected to overfitting. The maximum *mAP*0.5 value of 0.8881 was achieved at fold K5, and the minimum *mAP*0.5 value of 0.861 was achieved at fold K4.

The results of all the state-of-the-art models trained on the SHEL5K dataset are summarized in Table 8. The performance of the YOLO models was compared with the Faster-RCNN with the Inception V2 architecture. YOLOv3-tiny [10], YOLOv3 [9], and YOLOv3-SPP [11] were the models pretrained on the ImageNet dataset [32], while YOLOv5s, YOLOv5m, and YOLOv5x [13] were pretrained on the COCO128 dataset [31]. Detection results of the best yolov5x [13] models trained on SHEL5K dataset and other publicly available datasets [8,20,27,28] are illustrated in Appendix A. The best *mAP*0.5 of 0.8828 was achieved by the YOLOR [16] model with a precision, recall, and F1 score of 0.9322, 0.8066, and 0.8637, respectively. The lowest *mAP*0.5 score of 0.3689 was achieved by the Faster-RCNN [14] with a precision, recall, and F1 score of 0.7808, 0.3862, and 0.5167, respectively. The Faster-RCNN model achieved the highest inference time of 0.05 s. In the YOLO models, the YOLOv3-tiny [10] achieved the lowest *mAP*0.5 score of 0.3779 with a precision, recall, and F1 score of 0.7695, 0.4225, and 0.5408, respectively. Table 8 show the training time and testing time of all the models. The YOLOv3 tiny model had the lowest inference time of 0.006 s and fewer layers and parameters as compared to the other YOLO models. The YOLOR model achieved the highest *mAP*0.5 of 0.8828 with an optimum inference time of 0.012 s.

## 5. Conclusions

The proposed work aimed to extend the number of classes and labels of the publicly available SHD dataset [8]. The SHD dataset [8] contains 5000 images with three object classes (*helmet*, *head*, and *person*); however, most of the images were incompletely labeled. Therefore, a new dataset named SHEL5K (publicly available at https://data.mendeley.com/datasets/9rcv8mm682/draft?a=28c11744-48e7-4810-955b-d76e853beae5 (accessed on 5 January 2022)) was proposed by adding three more classes and completely labeling all 5000 images of the SHD dataset [8]. The proposed dataset was benchmarked on the various state-of-the-art one-stage object detection models, namely YOLOv3-tiny [10], YOLOv3 [9], YOLOv3-SPP [11], YOLOv4 [12], YOLOv5-P5 [13], the Faster-RCNN [14] with Inception v2 [15], and YOLOR [16]. The experimental results showed significant improvements in the *mAP*0.5 s of the compared models. From the experimental result of the models on the proposed dataset (SHEL5K), it can be concluded that all the models showed promising performances in detecting all classes. It can also be concluded that the proposed dataset had an advantage over the SHD dataset [8] in terms of images and labeling. Moreover, models trained on the proposed dataset can be used for a real-time safety helmet detection task. In the future, we will improve the real-time recognition rate of the safety helmet detection focusing on misclassified cases.

## Figures and Tables

**Table 1:** Comparison of public safety helmet datasets and the SHEL5K dataset.

Datasets	Hardhat [20]	HHW [27]	SHD [8]	SHW [28]	SHEL5K
Total sample	7063	7041	5000	7581	5000
Class	3	3	3	2	6
Number of labels in each class					
Helmet	19,852	26,506	18,966	-	19,252
Head	6781	8263	5785	-	6120
Person *	616	998	751	9044	-
Head and helmet	-	-	-	-	16,048
Person not helmet	-	-	-	-	5248
Person and helmet	-	-	-	-	14,767
Face	-	-	-	-	14,135
Hat **	-	-	-	111,514	-
Total	27,249	35,767	25,502	120,558	75,570

* The *person* class of the SHD dataset is called *head*. ** The *hat* class of the SHD dataset is called *helmet*.

**Table 2:** Comparison between two dataset results for 3 classes on the YOLOv3-SPP [11] and YOLOv5x [13] models.

	YOLOv3-SPP [11]							
	SHD Dataset [8]				SHEL5K Dataset with 3 Classes			
**Class**	**Precision**	**Recall**	***mAP*** **0.5**	**F1**	**Precision**	**Recall**	***mAP*** **0.5**	**F1**
Helmet	0.9578	0.4976	0.4869	0.6549	0.9222	0.7197	0.7028	0.8084
Head	0.9154	0.302	0.2923	0.4542	0.9114	0.6642	0.6484	0.7684
Person	0	0	0	0	0.9092	0.6354	0.6148	0.748
Average	0.6244	0.2665	0.2597	0.3697	0.9143	0.6731	0.6553	0.775
	**YOLOv5x [13]**							
	**SHD Dataset [8]**				**SHEL5K Dataset with 3 Classes**			
**Class**	**Precision**	**Recall**	***mAP*** **0.5**	**F1**	**Precision**	**Recall**	***mAP*** **0.5**	**F1**
Helmet	0.9559	0.9162	0.9162	0.9356	0.9402	0.8858	0.8774	0.9122
Head	0.909	0.879	0.8686	0.8938	0.9216	0.8562	0.8499	0.8877
Person	0.0345	0.0052	0.0003	0.009	0.9203	0.8409	0.8311	0.8788
Average	0.6331	0.6001	0.595	0.6128	0.9274	0.861	0.8528	0.8929

**Table 3:** Comparison results of different variations of the YOLOv3 models (a) trained from scratch, (b) pretrained on the ImageNet dataset [32], and (c) pretrained on the MS COCO dataset [31].

	YOLOv3-SPP [11]											
	Scratch				Pretrained on ImagesNet Dataset [32]				Pretrained on MS COCO Dataset [31]			
**Class**	**Precision**	**Recall**	***mAP*** **0.5**	**F1**	**Precision**	**Recall**	***mAP*** **0.5**	**F1**	**Precision**	**Recall**	***mAP*** **0.5**	**F1**
Helmet	0.9253	0.3144	0.3053	0.4693	0.9373	0.6275	0.6105	0.7518	0.8277	0.2971	0.2602	0.4372
Head with helmet	0.9244	0.4035	0.3871	0.5618	0.9349	0.6668	0.6459	0.7784	0.7806	0.463	0.4043	0.5813
person with helmet	0.7778	0.1442	0.12	0.2433	0.8746	0.6288	0.5924	0.7316	0.8622	0.4491	0.4076	0.5906
Head	0.8868	0.2295	0.2173	0.3646	0.9268	0.6184	0.5978	0.7418	0.8378	0.2775	0.2422	0.4169
Person without helmet	0.8241	0.1563	0.1339	0.2628	0.8784	0.4957	0.4729	0.6338	0.8389	0.3823	0.3528	0.5252
Face	0.4191	0.0556	0.0295	0.0982	0.7588	0.4715	0.4238	0.5816	0.3978	0.013	0.007	0.0252
Average	0.7929	0.2173	0.1988	0.3333	0.8851	0.5848	0.5572	0.7032	0.7575	0.3137	0.279	0.4294

**Table 4:** Comparison results of different variations of YOLOv5-P5 [13]: (a) YOLOv5s, (b) YOLOv5m, and (c) YOLOv5x.

	YOLOv5s [13]				YOLOv5m [13]				YOLOv5x [13]			
Class	Precision	Recall	*mAP*0.5	F1	Precision	Recall	*mAP*0.5	F1	Precision	Recall	*mAP*0.5	F1
Helmet	0.961	0.7825	0.872	0.8626	0.9632	0.7981	0.8795	0.8729	0.96	0.8205	0.8896	0.8848
Head with helmet	0.9437	0.7973	0.8761	0.8608	0.9476	0.7946	0.8783	0.8641	0.9357	0.8247	0.8912	0.8767
Person with helmet	0.9061	0.8385	0.8935	0.871	0.9131	0.8346	0.8922	0.8721	0.8953	0.8723	0.9089	0.8836
Head	0.9341	0.8219	0.889	0.8744	0.9335	0.8252	0.8897	0.876	0.9344	0.8497	0.9025	0.89
Person without helmet	0.8791	0.7583	0.8493	0.8142	0.8872	0.7602	0.8527	0.8188	0.8921	0.7924	0.8732	0.8393
Face	0.8991	0.6514	0.7863	0.7558	0.9061	0.6982	0.8122	0.7886	0.895	0.7427	0.8301	0.8117
Average	0.9207	0.774	0.861	0.8397	0.9251	0.7851	0.8687	0.84887	0.9188	0.817	0.8826	0.8644

**Table 5:** The result of the YOLOv5x [13] and YOLOR [16] models on the publicly available datasets and the proposed SHEL5K dataset.

		YOLOv5x [13]				YOLOR [16]			
Datasets	Class	Precision	Recall	*mAP*0.5	F1	Precision	Recall	*mAP*0.5	F1
SHW [28]	2	0.9334	0.9297	0.9219	0.9294	0.9486	0.8063	0.889	0.8697
Hardhat [20]	3	0.6715	0.6545	0.6389	0.6546	0.6367	0.6263	0.6407	0.6315
HHW [27]	3	0.6355	0.6295	0.6214	0.6288	0.6289	0.6177	0.6344	0.6233
SHD [8]	3	0.6331	0.6001	0.595	0.6128	0.6211	0.6341	0.6431	0.6276
SHEL5K	6	0.9187	0.817	0.8826	0.8644	0.9322	0.8066	0.8828	0.8637

**Table 6:** The result of the YOLOR [16] model on the SHEL5K dataset.

YOLOR [16]				
**Class**	**Precision**	**Recall**	***mAP*** **0.5**	**F1**
Helmet	0.9658	0.7981	0.8846	0.874
Head with helmet	0.9464	0.8172	0.8898	0.877
Person with helmet	0.9225	0.8771	0.9204	0.8992
Head	0.9461	0.8464	0.9068	0.8935
Person without helmet	0.8859	0.8019	0.8767	0.8418
Face	0.9264	0.6992	0.8182	0.797
Average	0.9322	0.8066	0.8828	0.8637

**Table 7:** The results of K-fold cross-validation on the SHEL5K dataset using the YOLOR model [16].

	K1		K2		K3		K4		K5	
	*mAP*0.5	F1	*mAP*0.5	F1	*mAP*0.5	F1	*mAP*0.5	F1	*mAP*0.5	F1
Helmet	0.8846	0.874	0.8813	0.8704	0.8878	0.8787	0.881	0.8702	0.8896	0.878
Head with helmet	0.8898	0.877	0.8848	0.8741	0.8932	0.8815	0.8859	0.8713	0.8953	0.88
person with helmet	0.9204	0.8992	0.9146	0.8976	0.9213	0.9048	0.9319	0.9117	0.9226	0.9037
Head	0.9068	0.8935	0.893	0.8805	0.8979	0.885	0.9068	0.8921	0.9134	0.9003
person without helmet	0.8767	0.8418	0.8731	0.8433	0.8867	0.8547	0.8749	0.8412	0.8832	0.8584
face	0.8182	0.797	0.8213	0.7943	0.814	0.79	0.8094	0.7795	0.8244	0.8008
Average	0.8828	0.8637	0.878	0.8614	0.8835	0.8658	0.8817	0.861	0.8881	0.8714

**Table 8:** Results of state-of-the-art models on the SHEL5K dataset.

Models	Precision	Recall	*mAP*0.5	F1	Training Time (hours)	Testing Time (s)	Parameters (Million)	Layers
Faster-RCNN [14]	0.7808	0.3862	0.3689	0.5167	55.6	0.084	13.3	48
YOLOv3-tiny [10]	0.7695	0.4225	0.3779	0.5408	5.2	0.006	8.7	37
YOLOv3 [9]	0.8509	0.4482	0.417	0.5848	24.6	0.011	61.6	222
YOLOv3-SPP [11]	0.8851	0.5848	0.5572	0.7032	24.6	0.012	62.6	225
YOLOv4 [12]	0.925	0.7798	0.7693	0.8449	11.2	0.014	63.9	488
YOLOv4_pacsp-x-mish_ [12]	0.9195	0.8036	0.7915	0.8567	14.5	0.014	63.9	488
YOLOv5s [13]	0.9205	0.774	0.861	0.8397	0.3	0.018	7.1	224
YOLOv5m [13]	0.9251	0.7851	0.8687	0.8488	2.7	0.022	21.1	308
YOLOv5x [13]	0.9188	0.817	0.8826	0.8644	6.3	0.032	87.2	476
YOLOR [16]	0.9322	0.8066	0.8828	0.8637	9.8	0.012	36.9	665

**Figure A1:**
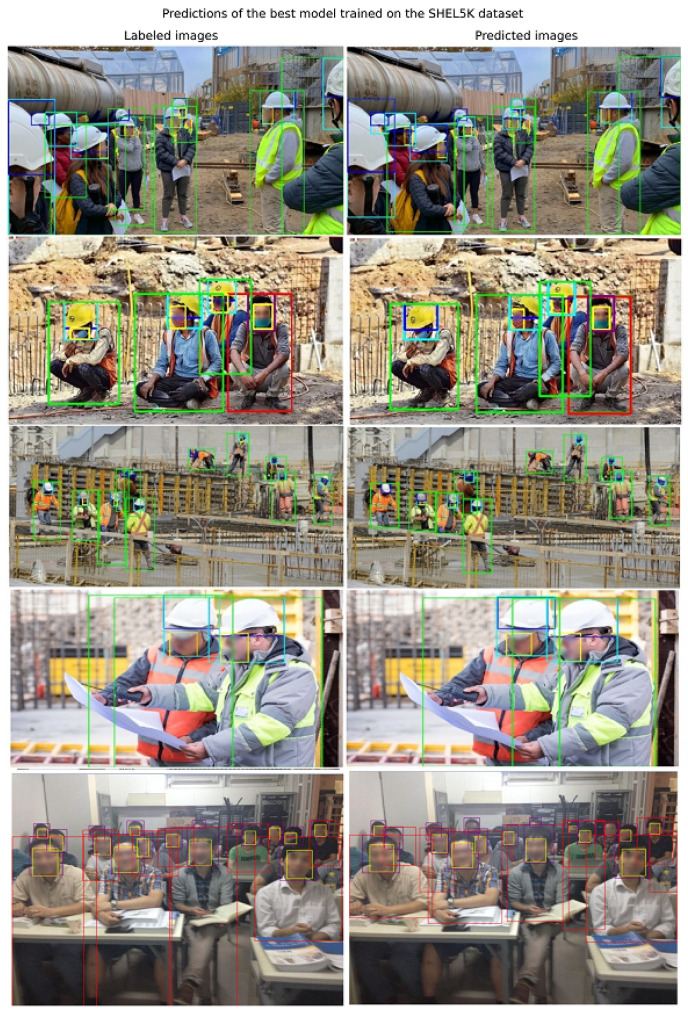
Predictions of the best model trained on the SHEL5k dataset and the ground truth labels of the sample images.

**Figure A2:**
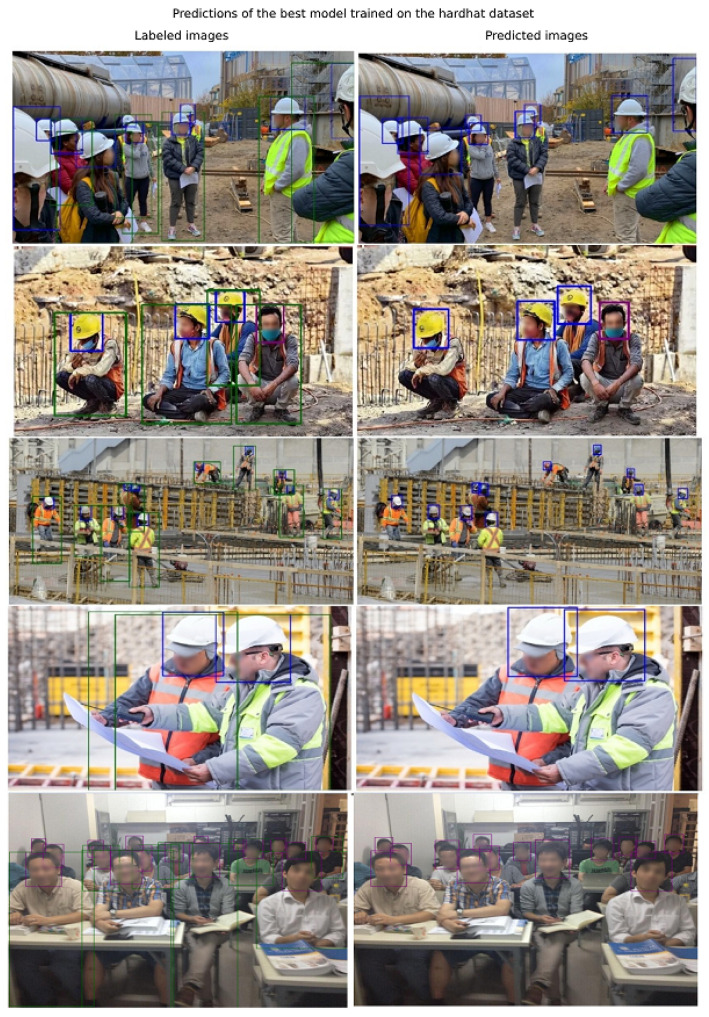
Predictions of the best model trained on the hardhat dataset [20] and the ground truth labels of the sample images.

**Figure A3:**
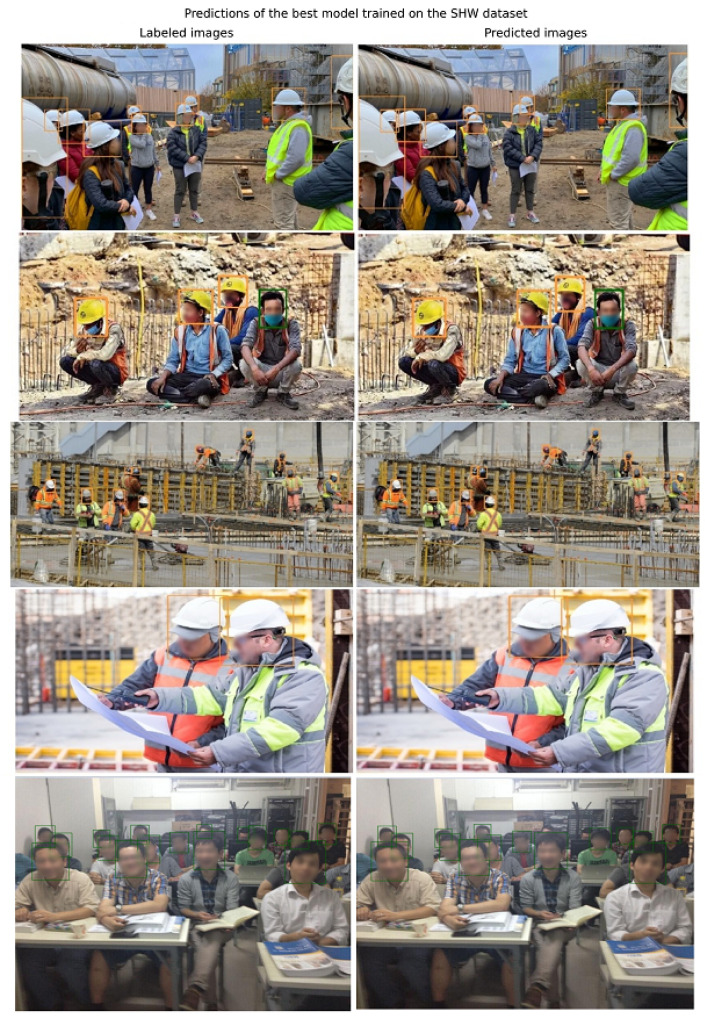
Predictions of the best model trained on the SHW dataset [28] and the ground truth labels of the sample images.

**Figure A4:**
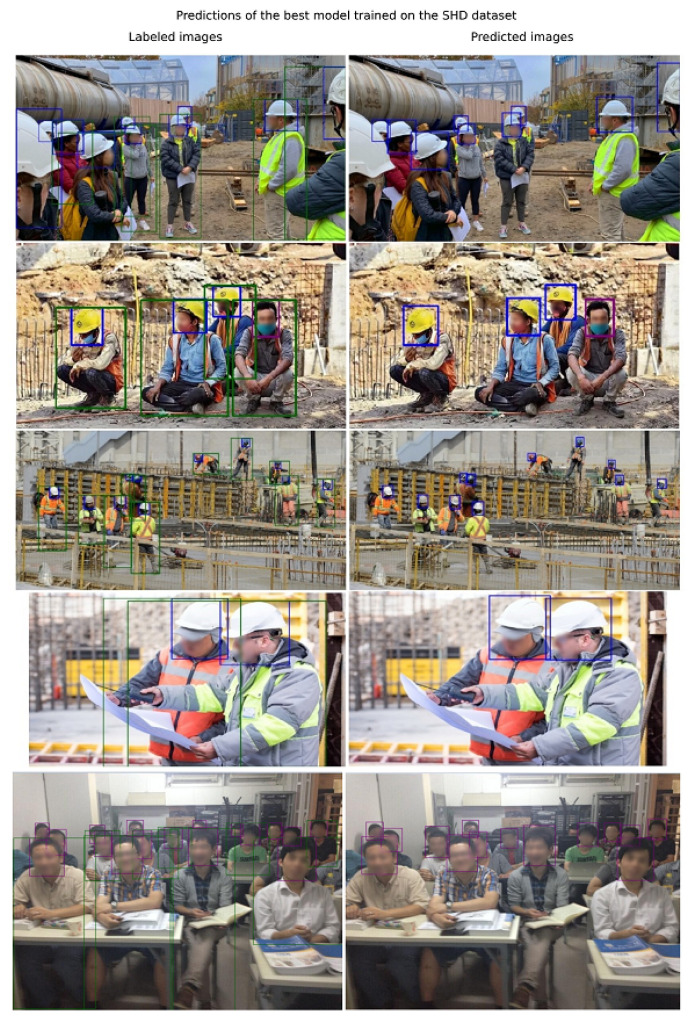
Predictions of the best model trained on the SHD dataset [8] and the ground truth labels of the sample images.

**Figure A5:**
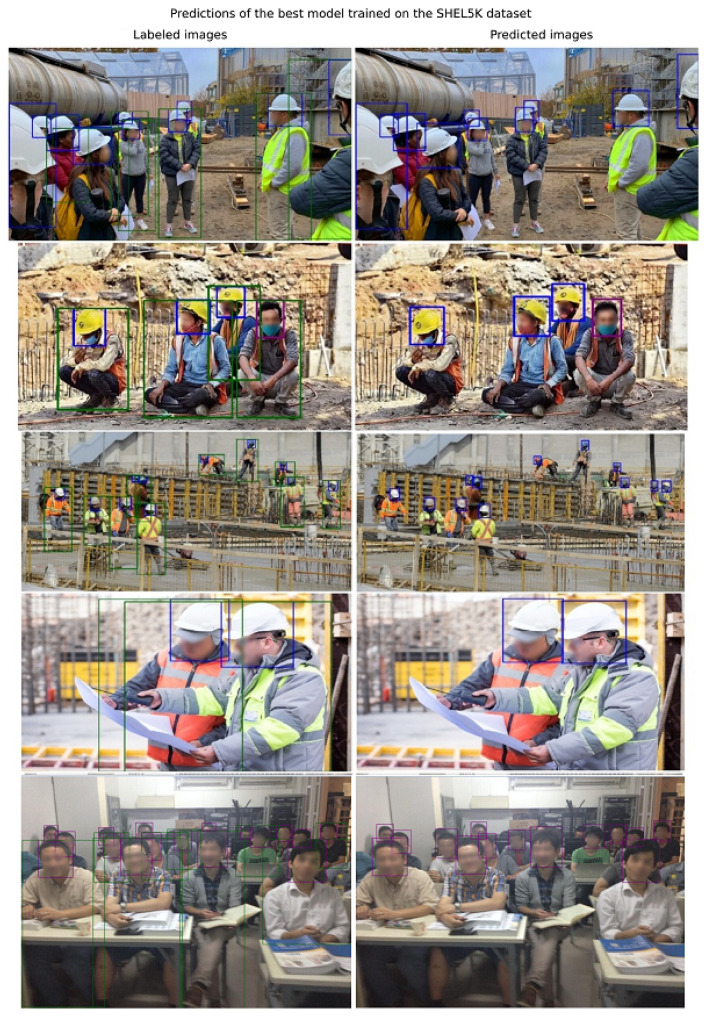
Predictions of the best model trained on the HHW dataset [27] and the ground truth labels of the sample images.

## Data Availability

The dataset is publicly available in Mendeley Data and can be found at: https://data.mendeley.com/datasets/9rcv8mm682/draft?a=28c11744-48e7-4810-955b-d76e853beae5 (accessed on 5 January 2022).
